# Tri-State Medical Society of Georgia, Alabama and Tennessee

**Published:** 1895-11

**Authors:** 


### Society Reports.

---

### TRI-STATE MEDICAL SOCIETY OF GEORGIA, ALABAMA, AND TENNESSEE.

### Seventh Annual Meeting.

#### FIRST DAY.

Dr. R. M. Cunningham, *President*, presiding.

Session opened with prayer by Rev. J. W. Bachman. After the transaction of miscellaneous business, the President delivered his address entitled

TUBERCULOSIS, based on a large experience in the penitentiary of Alabama.

The paper showed a wonderful predisposition of the negro race to tuberculosis as compared with the whites, which he explains as follows:

1st. The negro has acquired, since his emancipation, a predisposition to tuberculosis which is hereditary.

2d. A greater liability to diseases which produce a local predisposition to tuberculosis, especially bronchial and intestinal catarrh and pleurisy.

3d. He is physically and mentally and morally inferior to the white man, therefore,

4th. He is more liable to contract disease, particularly tuberculosis and thoracic diseases (local and from infection) generally.

5th. His changed social, religious, political, and industrial relations, involving, as a rule, a change from the segregate to the aggregate from country to town, and from farm to public works.

6th. His disregard for all rules of sanitation.

The greatest mortality was between twenty and thirty years. In two prisons, at Pratt City, a mile apart and under the same management and hygienic conditions, there were at No. 1, forty-five deaths from tuberculosis, at No. 2, twenty-six deaths. This was explained by the fact that there was an epidemic of diarrhœa at No. 1, proving that intestinal catarrh predisposes to tuberculosis of the peritoneal form, the excess of mortality at No. 1 over No. 2 being nineteen, exactly the number which died from tubercular peritonitis. He always found an epidemic of diarrhœa followed by a large number of cases of tubercular peritonitis.

The cause of tuberculosis he defined to be, first, essential, the tubercle bacillus; second, the predisposing, (*a*) an inherited constitutional predisposition, or (*b*) an acquired constitutional or local predisposition. The predisposing never, alone, produces the disease, the essential rarely. Primarily, it is the result of hetero-inoculation and, as a rule, is a local infection producing chronic local changes, the general and acute forms being due to auto-infection.

In general, acute miliary tuberculosis, the lungs, peritoneum, spleen, sometimes the liver, rarely the kidneys, were involved, the patient dying before the caseous stage of the disease set in. These cases follow pleurisy frequently, notably after a large amount of fluid was withdrawn and from tubercular glands. In the chronic general forms, the process involves the same organs, but the infection is not so general and the caseous stage is often reached and local inflammatory lesions generally found. In both there may be an active tubercular inflammation developed in some organs, rapidly terminating the disease. The bacilli were distributed by the circulation.

The acute forms are generally the result of auto-infection from a chronic tuberculosis, which may not have been suspected. The acute forms are very rare. The bone is rarely affected, the one most frequently involved being the sternum.

H. BERLIN thought that the reason the disease prevailed so largely in prisons was, first, because the convicts were generally in a weakened condition; second, while the buildings were apparently clean, still, they contained the germs; but the most important was the fact that the dust inhaled, which shows sharp corners under the microscope, cut the delicate tissues and admitted the bacilli.

C. HOLTZCLAW had noticed the frequency of tuberculosis in the county institution. While the left lung was first affected, in the white, it was the right, in the negro. The greater liability of the negro to the disease was due to the fact that the white race acquired an immunity from long contact.

J. B. MURFREE could not endorse that diarrhœa had anything to do with inducing the disease, except as it weakened the patient. While the bacillus was the cause, an equally important factor was the predisposition.

J. B. COWAN thought we could lift up the vital energy and put the functional activity in such a condition as to resist the disease.

G. A. BAXTER differed with Dr. Murfree in regard to diarrhœa as a factor in the production of the disease. It acts by allowing an entrance for the bacilli. One agent the writer did not mention was the food, and especially milk. In China, where the disease is unknown, it is attributed to the nonuse of milk. He had frequently seen tuberculosis in bones and in the glands of the neck.

He could see no objection to the removal of the latter. An imperfect removal might result in general infection, but where there are two or three they could be thoroughly removed.

P. L. BROUILLETTE regretted that the microscope had not been used by the writer, as it was sometimes impossible to diagnose tuberculosis.

Dr. CUNNINGHAM, in closing the discussion, said that dust produced a fibroid phthisis, but did not produce tuberculosis, but might predispose to the disease. He did not find typical tuberculosis in the negro. Believes he is acquiring a predisposition to tuberculosis. Both lungs are affected, as a rule. The diarrhœa predisposed to the disease by abrading the membrane and giving entrance to the bacilli. The weakness of the paper was that the microscope was not used, but the clinical history and the gross lesions were enough to complete the diagnosis.

G. MANNING ELLIS, of Chattanooga, read a paper,

PSEUDO-HYPERTROPHIC MUSCULAR PARALYSIS, and presented a patient, giving the characteristic history. The boy was late in attempting to walk. The muscles were large, and they seemed well developed. The gait was oscillating, the child, while it seemed healthy and well developed, showed an increase in the difficulty of locomotion. The characteristic symptoms were shown. The peculiar gait, the lardosis, the manner of rising by placing the hands on the knees and then on the thighs, climbing up, as it were, with his hands, which is almost pathognomonic, the absence of the tendon reflex, and the diminution of the muscles of the legs which comes after the enlargement. The upper extremities showed an enlarged infraspinatus and the decrease in size of the latissimus dorsi, producing an absence of the axillary fold.

WILLIS F. WESTMORELAND asked if there was any adherent prepuce. The reply was that there was not. He reported two cases in which there was an adherent prepuce. In one there was an operation in the late stage, but with no improvement. He thought that if there were irritation from this that an operation before the disease began might be of benefit.

NIGHT SESSION.

A reception was tendered the Society at the Southern Sanitarium by Dr. and Mrs. R. P. Johnson.

SECOND DAY.

J. B. MURFREE, of Murfreesboro, Tennessee, read a paper on

THE PLACENTA, HOW AND WHEN DELIVERED.

He thought as soon as the child was born, Crede's method should be employed. If the placenta does not come away in twenty minutes, gentle traction should be made on the cord. Undue force should not be used. If this does not succeed, and especially if the placenta presents centrally, the hand should be introduced and the edge freed from its attachments. If the placenta was delivered as soon as the child was born, it would leave the mouths of the vessels open. Exactly when to remove it cannot be definitely stated in every case. Most practitioners wait too long.

R. R. KIME took issue with the author in regard to introducing the hand into the uterus, as that would add greatly to the danger of infection, and was rarely necessary. By wrapping the cord around the fingers of the right hand and pressing back the posterior lip of the cervix, the placenta will slip out. He protested against the use of ergot, which contracted the os and prevented the delivery of the placenta.

W. G. BOGART delivers the placenta as soon as he ties the cord and gives the child to the nurse and prepares his hands, which takes about twenty minutes. He grasps the fundus and squeezes the placenta out if possible; if not successful, he introduces two fingers into the uterus and makes a rotatory motion, with the other hand on the fundus. Never makes contraction on the cord, which he deems dangerous. Ergot should not be given to expel the placenta. The important point is to get all the placenta away. He has no fear of putting the hand into the uterine cavity if it is thoroughly cleansed, nor does he deem it necessary, for this reason, to use intrauterine douche.

PRESTON SCOTT thought we were all too hasty to get rid of the placenta. Time should be given for tonic contraction. Gentle tractions should be made on the twisted body of the placenta. Had excluded ergot from his practice entirely. He used the hot water douche to promote contraction.

J. P. STEWART waits longer than twenty minutes, an hour if there was no hemorrhage. If the placenta was in the vagina, delivers at once, as it acts as a foreign body, causing contraction and tearing the membranes.

J. B. COWAN thought there was a happy medium between twenty minutes and an hour. He assists nature during the pains. If necessary, puts his hand into the uterus. Post partem hemorrhage can be prevented by steady pressure on the fundus, kept up an hour if necessary. Never pulls the cord.

Dr. MURFREE said that ergot was liable to produce hourglass contraction. There can be no harm in introducing the hand into the uterus.

E. H. SHOLL, of Birmingham, read a paper entitled

### REFLECT, reporting cases which were not relieved because not properly investigated. A case treated for liver disease, was relieved by treatment indicated by an examination of the urine; one diagnosed as consumption, relieved by a similar course; one of headache was found due to diabetes.

In these cases, no examination of the urine had been made. Doctors, as a rule, should study their cases more.

J. B. COWAN said that the lesson of the paper was that all should learn to think. In this respect, he found fault with the modern education, which was too much a process of cramming.

WILLIS F. WESTMORELAND thought that the modern medical college taught that every patient should be examined as well as if for life insurance. The day when albumen could be seen by looking at the bottle, was passed. There was no college of any pretensions which did not teach examination of urine and the use of the microscope. The modern student was better qualified than those of the past.

J B. MURFREE thought the point of the paper was that we did not take time to think about our cases, not that we did not know how to make these examinations. The facts were not properly put together.

PRESTON SCOTT was surprised that in any cases seen in consultation, no examination of the urine had been made and the cases not properly studied.

W. C. TOWNES asked the writer what he meant by a rigid diet. Dr. Sholl said that he meant the exclusion of all starchy foods, the use of meat, milk, eggs, oysters, cheese, turnip greens and such articles.

WILLIS F. WESTMORELAND presented

### REPORT OF CASES (a) TRACHEOTOMY FOR FOREIGN BODIES, (b) CYSTOTOMY FOR STONE.

He reported twenty-seven successful cases of tracheotomy for foreign bodies. In all, the first ring was cut and the opening enlarged downward as necessary. Operated as early as possible. The external incision was made large. Muscles were separated, the fascia divided, vessels litigated, so that no forceps were in the road. If necessary, the isthmus of the thyroid was divided and litigated. No tenaculum was used in the trachea.

Instead of trachea forceps, the cut was held open with silk thread, introduced with a needle having no cutting edge. The foreign body was not probed for, but the wound left open, if necessary, in one case (cocklebur) for three days. The mucus will sometimes prevent the closure of the wound. In closing the latter, the tissue under the mucous membrane is brought up together with the silk, using a needle having no cutting edge and the Halsted or mattress suture. Thus the layers are all brought together.

In cystotomy for stone, he avoids rectal dilatations. To distend the bladder, he uses hydrostatic pressure, raising the water two feet. This causes the bladder to bulge through the opening. No tenaculum is used to steady the bladder, but it is held with two artery forceps, between which the opening is made as high as possible. After removing the stone and flushing the bladder, the opening is closed with the Halsted suture. The closure is tested by raising the water, for an instant, three feet.

G. A. BAXTER endorsed the position of the author. He was attached to the medio-lateral operation. Stone is rare in this section, there not having been a dozen operations in fifteen years. The disease is more frequent in Middle Tennessee. He advocated drainage in the suprapubic operation.

J. A. GOGGANS related a case in which the larynx at the site of the thyroid cartilage was almost occluded by a new growth so that he had to operate rapidly to save the patient. Throwing the head back increased the difficulty of breathing and the head had to be held forward and the larynx steadied with a tenaculum. He said that the suprapubic was the operation, par excellence, for stone. He was prejudiced against the low operations, as he was once called on to sew up a large fistulous opening between the bladder and the rectum in a case sent him. He had attempted to repair this opening by silkworm gut, operating through the rectum. At the third operation, he had divided the sphincter ani muscle and almost closed the fistula.

W. E. B. DAVIS said that he was glad that both operations were fairly considered. The results of the older surgeons who performed the low operations were good. He found that statistics of the suprapubic operations were bad because bad cases were selected for that operation. A distinguished surgeon had so operated to save his statistics for the low operation. This shows how statistics can be doctored. He thought it well to leave the wound open for drainage for there is always disease of the bladder, and we may have a new stone in a few weeks.

R. J. TRIPPE said that his experience was limited to a few cases operated on for disease, and advocated leaving the wound open for drainage. As a guide, he used a sound and did not fill the bladder, but always washed it out before the operation. In tracheotomy, he used long scissors forceps and did not stop to litigate the vessels. These held the wound open and saved one assistant.

Dr. WESTMORELAND, in closing the discussion, said that he used no instrument to hold the trachea open, as that took some room, while the silk did not. Time was an important item, but we always have two minutes after the child quits breathing, as he can be resuscitated after that. He advocated cutting high in the suprapubic operation, as the bladder is near the surface, being quite deep near the pubis. The tenaculum was avoided, as the urine might be voided through the small opening. The buried silver wire suture was used, owing to the fact that it took several weeks for the muscular tissues to unite, and no other structure would last that long. Without this suture, according to the statistics of Greig-Smith, twenty per cent. of cases of laparotomy had hernia. In the high operation, it would make no difference if the peritoneum were cut. The wound, in these cases, was not left open, as they were not cases of disease.

J. A. GOGGANS, of Alexander City, Alabama, read a paper entitled

### EARLY DIAGNOSIS AND VAGINAL HYSTERECTOMY IN CANCER OF THE UTERUS, dwelling on the importance of prophylaxis, early diagnosis and early operative treatment by vaginal hysterectomy.

*(To b. continued.)*

